# Analysis of the Results of Cytomegalovirus Testing Combined with Genetic Testing in Children with Congenital Hearing Loss

**DOI:** 10.3390/jcm11185335

**Published:** 2022-09-11

**Authors:** Yuan Jin, Xiaozhou Liu, Sen Chen, Jiale Xiang, Zhiyu Peng, Yu Sun

**Affiliations:** 1 Department of Otorhinolaryngology, Union Hospital, Tongji Medical College, Huazhong University of Science and Technology, Wuhan 430022, China; 2 College of Life Sciences, University of Chinese Academy of Sciences, Beijing 100049, China

**Keywords:** cytomegalovirus, congenital hearing loss, screening, genetic testing

## Abstract

To improve the etiological diagnosis of congenital hearing loss by combining whole-exome sequencing (WES) with cytomegalovirus (CMV) testing and to explore the potential benefits of adding CMV screening to newborn hearing screening, 80 children under 2 years of age with bilateral sensorineural hearing loss were recruited. Peripheral venous blood was extracted from the children for WES analysis. Saliva after mouthwash and the first urine in the morning were collected and used as samples to quantify CMV DNA copy number in urine and saliva by qPCR; among the 80 children with congenital deafness, 59 (74%) were found to have genetic variants that may cause congenital deafness, including 44 with *GJB2* or *SLC26A4* gene variant, 1 with *STRC* gene variant, and 14 with other genetic variants. A total of 12 children carried deafness gene variants associated with a syndrome; CMV test results showed that in two children, the CMV DNA copy number in saliva was >1000/mL, which indicates that they were CMV-positive, and their genetic test results were negative. A neonatal CMV test combined with genetic screening can improve the etiological diagnosis rate of congenital deafness, and the direct evidence of neonatal CMV infection deserves further verification.

## 1. Introduction

Congenital hearing loss is the most common sensory deficit in children. It has been reported that 1–2 per 1000 newborns have sensorineural hearing impairment [1]. More than 50% of cases of congenital hearing loss in children are attributed to genetic aetiologies [2]. To date, 218 genes causing deafness have been identified [3]. Among the non-genetic factors associated with congenital hearing loss, cytomegalovirus (CMV) infection is one of the most common, accounting for 10–20% of cases of sensorineural hearing impairment reported in children [4]. Countries and regions with different economic conditions have different rates of neonatal CMV infection. In general, the infection rate of human CMV in live newborns is 0.3–2.4% [5]. In developing countries, the incidence can reach 1–5% of all live births [6].

CMV mainly invades the liver, followed by the brain, bone marrow, lung, heart, and other organs, which can lead to neonatal birth defects and developmental disorders [7]. Approximately 85–90% of infected newborns have no symptoms at birth, and 10–15% of infected newborns have obvious symptoms of clinical infection [8]. Clinical manifestations of CMV include foetal growth restriction, low birth weight, central nervous system and multiple organ involvement with petechiae, hepatomegaly, splenomegaly, jaundice, pneumonia, and encephalitis [9]. These manifestations are severe and can cause a high perinatal mortality rate and major neurological sequelae in approximately 90% of surviving infants with symptomatic CMV [10]. In addition, 10–15% of infants with asymptomatic CMV develop long-term sequelae, including progressive sensorineural hearing difficulty and mental retardation [11]. These sequelae add to the complexity of the diagnosis of CMV infection.

If children with sensorineural hearing loss do not receive timely intervention, the development of their speaking ability, intelligence, and learning ability is seriously affected, which substantially impacts their individual development and future. Although newborn hearing screening is universal in most developed countries, screening for CMV in newborns is not widespread [12]. Determining whether congenital CMV infection or genetic factors are the cause of hearing loss in these newborns is important for early diagnosis and intervention. To improve the aetiological diagnosis rate of congenital deafness, we recruited children with congenital sensorineural disease and performed deafness genetic testing and CMV testing. Due to the high cost of whole-genome sequencing, we adopted a low-cost comprehensive genetic testing protocol. In this study, we identified several children who were CMV-positive via CMV screening and did not carry genetic variants that cause deafness. Although the CMV test results did not diagnose congenital CMV infection in these children, we considered their congenital hearing loss to be linked to congenital CMV infection based on their clinical symptoms. To more accurately diagnose the aetiology of congenital deafness, provide a more optimized diagnosis and treatment strategy, and improve the quality of life of children with congenital CMV, we propose adding high-sensitivity and high-specificity CMV screening to newborn hearing screening.

## 2. Materials and Methods

### 2.1. Clinical Examination

We recruited 80 children with bilateral hearing loss to this study. All participating children failed newborn hearing screening. Based on newborn hearing screening results, auditory steady-state response results, and medical history, these participants were diagnosed with congenital hearing loss. The children were 24 months of age or younger at the time of the testing. All children were physically examined, and their medical history was collected to identify other systemic disorders or abnormalities.

### 2.2. Variant Detection and Analysis

The *GJB2* and *SLC26A4* genes were first analysed by a multiplex polymerase chain reaction (PCR) amplicon sequencing assay. Low-pass genome sequencing was then used to analyse *GJB6* gene deletion. After excluding the aforementioned genes, *STRC/OTOA* analysis was performed using a kit (SALSA^®^ MLPA^®^ P461 DIS probe mix kit, MRC-Holland, Amsterdam, the Netherlands). Patients suffering from severe or profound hearing loss or who had negative results from *STRC/OTOA* analysis were further referred for exome sequencing. Exome sequencing was performed using KAPA HyperExome Probes (Roche, Pleasanton, CA, USA) accompanied by 100 bp paired-end sequencing on an MGISEQ-2000 platform (BGI-Wuhan, Wuhan, China). All detected sequence variants were confirmed via Sanger sequencing (single nucleotide variants), quantitative PCR (qPCR; exon-level copy number variations (CNVs)), or low-pass genome sequencing (subchromosomal CNVs). The sequencing results were all aligned to the reference human genome (hg19) using the Burrows–Wheeler Aligner (BWA) Multi-Vision software package. According to the sequencing results of two probands, Sanger sequencing was performed to confirm whether their parents had the same mutations. All SNVs and indels were referenced and compared with multiple databases, including the National Centre for Biotechnology Information (NCBI) GenBank database (https://www.ncbi.nlm.nih.gov/nuccore, accessed on 6 January 2021), the Database of Single Nucleotide Polymorphisms (dbSNP) (http://www.ncbi.nlm.nih.gov/projects/SNP, accessed on 6 January 2021), and the 1000 Genomes Database (https://www.internationalgenome.org, accessed on 6 January 2021).

### 2.3. Test for CMV Infection

Quantitative detection of CMV DNA content was performed by BGI Genomics (Wuhan, China). A total of 0.2 mL saliva and 2 mL urine was collected from each patient participating in the study for the detection of CMV infection. Specimens were stored at 4 °C until they were processed in the virology laboratory. We used 0.2 mL of urine or saliva to prepare DNA for quantitative detection of CMV DNA content by real-time fluorescence qPCR. A CMV test was considered positive when the saliva or urine samples were confirmed to be positive for CMV DNA (CMV DNA ≥ 1 × 10^3^ copies/mL). Term infants with serum total bilirubin >220 μmol/L and preterm infants with serum total bilirubin >256 μmol/L were diagnosed with pathological neonatal jaundice. 

## 3. Results

### 3.1. Rate of CMV Infection

Of the 80 children with congenital bilateral hearing loss in the study, 59 (74%) were found to have genetic variants that may cause congenital deafness, including 44 with *GJB2* or *SLC26A4* gene variant, 1 with *STRC* gene variant, and 14 with other genetic variants that may cause deafness. No genetic variants that can cause deafness were detected in the remaining 21 children. Among the 59 cases of children carrying a gene variant that may cause deafness, 12 children carried deafness gene variants associated with a syndrome; however, symptoms in addition to hearing loss were found in only five children upon further examination, and no signs or symptoms associated with a syndrome were observed upon subsequent clinical examination of the remaining seven children. CMV test results revealed that in two children, the CMV DNA copy number in saliva was >1000/mL, indicating that they were CMV-positive, and their genetic test results were negative, with no variants identified in genes that are known to cause deafness. The medical history of these two children included symptoms of congenital CMV infection, such as hyperbilirubinemia, ventricular defects, and patent ductus arteriosus. We summarise the information of all children with CMV-positive or CMV-false positive results in Table 1.

### 3.2. Clinical Data

Auditory Brainstem Response (ABR) was used to detect the hearing thresholds of the subjects. For Subject 2, the hearing threshold of the left ear was 55 dB, and the hearing threshold of the right ear was 60 dB; for Subject 6, the hearing threshold of the left ear was 100 dB, and the hearing threshold of the right ear was 90 dB. The pure-tone average was categorised as normal, mild (26–40 dB), moderate (41–55 dB), moderately severe (56–70 dB), severe (71–90 dB), or profound (>90 dB).

**Figure 1:**
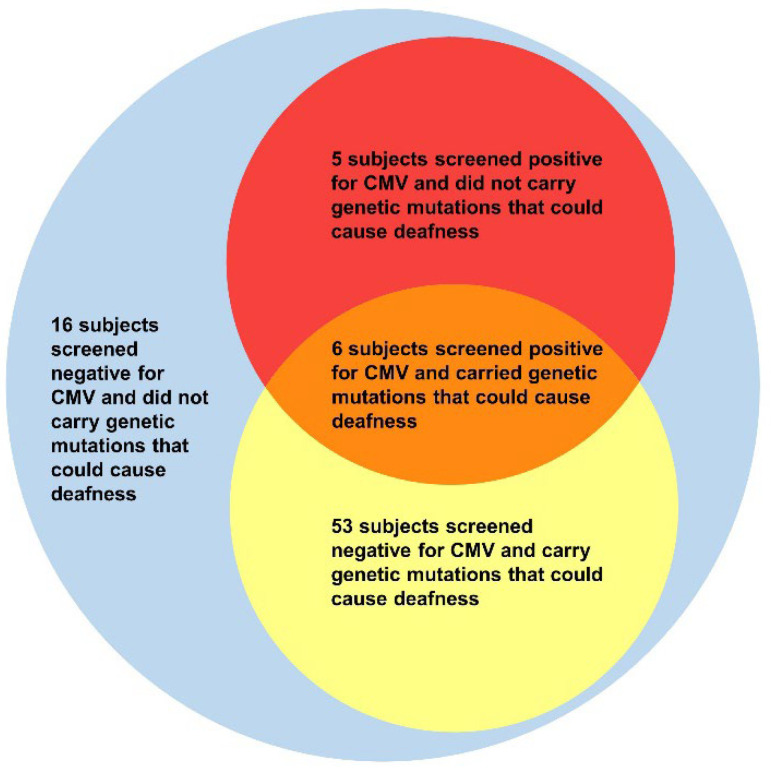
Graph of CMV screening and next-generation sequencing results.

Subject 2 suffered from bilateral congenital deafness. The parents of Subject 2 stated that Subject 2 was born with neonatal hyperbilirubinemia, neonatal pneumonia, and hypoalbuminemia. These were verified in Subject 2’s medical history and examination. Subject 6 was born with congenital heart disease (congenital patent ductus arteriosus). These are symptoms that may be observed in children with congenital CMV infection (Figure 1).

## 4. Discussion

Numerous studies have reported that CMV infection can cause congenital SNHL in children [13]; however, newborn CMV screening has not yet been established in routine practice in most countries. TORCH tests are performed on women of childbearing age in countries such as China [14]. Notably, many women around the world of reproductive age are seropositive for CMV [15]. In addition, it has been reported that women who are seropositive for CMV antibodies and may be immune may carry CMV-infected infants who are also at significant risk of congenital SNHL [16].

In our study, we collected blood, saliva, and urine samples and clinical data from 80 children who failed newborn hearing screening and attempted to diagnose these children with congenital deafness via next-generation sequencing and CMV screening. All 80 children were under 2 years of age at the time of the study. According to previous reports, the detection rate of CMV in newborns’ serum samples is lower than in saliva and urine samples. Serum samples were found to be positive in less than 40% of CMV-infected neonates. This suggests that saliva and urine samples are more suitable for newborn CMV screening. Saliva has a high detection rate but is prone to false-positive results; therefore, further testing on urine samples must be conducted in patients with CMV-positive saliva. In this study, CMV was detected in the saliva samples of 13 children and urine samples of 11 children. However, we considered children to be positive for CMV infection only when the CMV copy number detected in saliva or urine samples was >1000/mL. As shown in Table 1, there were two CMV-positive cases. Whole-exome sequencing revealed that they did not carry genetic variants that could cause hearing loss. In addition, the two children had a history of other clinical symptoms similar to those caused by congenital CMV infection. We believe that their congenital hearing loss may have been caused by congenital CMV infection.

In our study, genetic factors were found to account for 59/80 (74%) cases of congenital deafness. The proportion of children who are CMV-positive and do not carry the gene variant site is 0.025%. This value is much lower than the infection rate of CMV in children with SNHL [4,5]. This may be because the children we selected were all born with SNHL and were younger than 2 years of age. However, a considerable proportion of the hearing loss caused by congenital CMV infection is delayed hearing loss. Children who tested positive for CMV may have had a congenital infection, perinatal infection, or postnatal infection. However, we were not able to pinpoint the timing of CMV infection in the children. Moreover, we can only determine that these children had CMV infection; we cannot conclude whether their congenital hearing loss is related to CMV infection. As a result, diagnosis and treatment of these children is difficult. Notably, no association between urine or saliva CMV viral content and hearing loss severity was observed in our study. Of course, CMV virus levels in urine or saliva do not reflect the situation in the cochlea.

Early diagnosis and provision of antiviral therapy can help children with CMV infection lead normal lives. The efficacy of antiviral therapy in neonates with isolated SNHL due to CMV remains controversial. Because the replication of CMV in patients rebounds immediately after cessation of antiviral therapy, and SNHL in infants with CMV is often delayed; researchers question whether short-term antiviral therapy would have long-term benefit in the progression of SNHL [17]. However, it is worth noting that some studies have shown that infants born with isolated SNHL due to CMV were found to benefit from prolonged antiviral treatment. Marian G Michaels et al. treated nine symptomatic CMV-infected children with chronic intravenous or oral ganciclovir, seven had no progression of hearing loss, and two had improved hearing thresholds [18]. The placebo-controlled trial of valganciclovir therapy in neonates with symptomatic congenital CMV disease by David W Kimberlin et al. found that total-ear hearing (i.e., hearing in one or both ears that can be evaluated) was more likely to be improved or to remain normal at 12 months in the 6-month valganciclovir therapy group than in the 6-week valganciclovir therapy group [19]. The improvement in total-ear hearing was maintained at 24 months. In a retrospective study, Yehonatan Pasternak et al. found that infants born with isolated SNHL due to CMV showed significant improvement in hearing status and no deterioration of unaffected ears at baseline after receiving long-term antiviral therapy (intravenous ganciclovir 5 mg/kg/d for 6 weeks followed by oral valganciclovir or oral valganciclovir 17 mg/kg/dose in two daily doses for 12 weeks, then one daily dose until completion of 12 months of treatment) [20]. Although these antiviral treatments increase the risk of neutropenia, the benefit of long-term antiviral therapy on hearing in congenital CMV-infected patients deserves attention [19,20]. Antivirals should be started in the first month of life, and therapy must be based on a definitive diagnosis of CMV infection [21]. Therefore, screening for CMV in neonates is valuable.

Numerous studies have reported CMV screening combined with genetic testing for deafness in newborns who did not pass newborn hearing screening tests [22]. However, newborn CMV screening is not yet routinely performed in most countries. Foreign studies have shown that 68.3% of pregnant women have positive serum CMV IgG, of whom approximately 0.8% have active CMV infection [23]. In the United States, approximately 20,000 to 30,000 newborns are born with congenital CMV infection each year, of whom approximately 10–15% will suffer hearing impairment in childhood [24]. In China, approximately 95.3% of pregnant women are serum CMV-IgG positive [25]; among them, 3.5% were found to have acute or active CMV infection. Most newborns with congenital CMV infection are asymptomatic. We emphasise that newborn CMV screening is essential to identifying the aetiology of congenital deafness in newborns. The lack of newborn screening for CMV makes the diagnosis of congenital deafness difficult. Furthermore, we also propose including CMV screening in newborn hearing screening. However, there are problems in neonatal CMV screening that should be taken into consideration.

At present, there are no technical difficulties in the general screening for CMV in newborns, and a variety of samples and methods have been used to detect CMV. CMV screening of neonatal saliva samples by CMV PCR assay and collection of neonatal urine samples for further confirmation by PCR is an efficient and convenient method. However, widespread investigations have substantial economic costs. We can further reduce the risk of neonatal congenital CMV infection by developing CMV vaccines and reducing the exposure risk of women of childbearing age; however, ambiguity in diagnosis can lead to untimely interventions and antiretroviral treatment, causing children with SNHL to miss the optimal period of treatment and seriously hinder their healthy growth and development. We should calculate and balance the economic costs and social benefits of universal neonatal CMV screening. Studies have reported the potential cost-effectiveness and health benefits of universal screening among Chinese newborns [12]. Given that congenital CMV infection can cause progressive hearing loss, follow-up medical services such as hearing tests, examinations for other systemic diseases, and mental health counselling should also be provided, which increases the burden on the medical system. In any case, we need to set goals and create detailed plans to act as soon as possible.

## 5. Conclusions

Neonatal CMV testing combined with genetic screening can improve the aetiological diagnosis rate of congenital deafness, and direct evidence of neonatal CMV infection warrants further verification.

## Figures and Tables

**Table 1:** Information on all children with CMV-positive or CMV-false positive results.

Subject	Age at the Time of Sample Collection (Months)	CMV DNA Quantification in Saliva Sample	CMV DNA Quantification in Urine Sample	Variants in Congenital Hearing Loss–Related Genes
1	4	413,976.2	48,028.8	*GJB2* c. 560_605dup/c. 235del C
2	7	114,985.0	negative	-
3	6	93,588.6	negative	*SLC26A4* c. 919-2 A > G/c. 589 G > A
4	7	90,400.3	60,704.0	*GJB2* c. 263 C > T
5	8	48,376.9	6928.1	*KCNQ1* c. 1684 A > GT/EXON 8-9 DEL
6	7	37,786.3	negative	-
7	3	14,382.9	negative	*GJB2* c. 299_300 del AT/c. 235del C
8	21	negative	negative	-
9	18	negative	negative	-
10	22	negative	negative	*GJB2* c. 235del C/c. 235del C
11	12	negative	negative	*MYO7A* c. 6320 G > A/c. 612 C > G
12	22	negative	negative	-
13	20	negative	negative	*GJB2* c. 235del C/c. 176_191del

## Data Availability

The data used and analysed during the current study are available from the corresponding author on reasonable request. The data are not publicly available due to privacy or ethical restrictions.
